# Individual level microbial communities in the digestive system of the freshwater isopod *Asellus aquaticus* : Complex, robust and prospective

**DOI:** 10.1111/1758-2229.13142

**Published:** 2023-02-13

**Authors:** Aijuan Liao, Hanna Hartikainen, Claudia C. Buser

**Affiliations:** ^1^ ETH Zürich, Institute of Integrative Biology (IBZ) Zürich Switzerland; ^2^ Department of Ecology and Evolution University of Lausanne Lausanne Switzerland; ^3^ School of Life Sciences University of Nottingham, University Park Nottingham UK; ^4^ eawag, Swiss Federal Institute of Aquatic Science and Technology Dübendorf Switzerland

## Abstract

The freshwater isopod *Asellus aquaticus* is an important decomposer of leaf detritus, and its diverse gut microbiome has been depicted as key contributors in lignocellulose degradation as of terrestrial isopods. However, it is not clear whether the individual‐level microbiome profiles in the isopod digestive system across different habitats match the implied robust digestion function of the microbiome. Here, we described the bacterial diversity and abundance in the digestive system (hindgut and caeca) of multiple *A. aquaticus* individuals from two contrasting freshwater habitats. Individuals from a lake and a stream harboured distinct microbiomes, indicating a strong link between the host‐associated microbiome and microbes inhabiting the environments. While faeces likely reflected the variations in environmental microbial communities included in the diet, the microbial communities also substantially differed in the hindgut and caeca. Microbes closely related to lignocellulose degradation are found consistently more enriched in the hindgut in each individual. Caeca often associated with taxa implicated in endosymbiotic/parasitic roles (Mycoplasmatales and Rickettsiales), highlighting a complex host–parasite–microbiome interaction. The results highlight the lability of the *A. aquaticus* microbiome supporting the different functions of the two digestive organs, which may confer particular advantages in freshwater environments characterized by seasonally fluctuating and spatially disparate resource availability.

## INTRODUCTION

Bacterial symbionts represent one of the key drivers that can shape the ecology and evolution of their hosts and may ultimately coevolve with their hosts. Plant‐derived low‐nutrient diets of shredders and detritivores require specialized adaptations to regulate handling and feeding, excretion and nutrient assimilation and the production of digestive enzymes (Fenoy et al., 2020). Degradation of plant polysaccharides involves a large number of Carbohydrate‐Active enZymes (CAZymes) and no animal can achieve efficient digestion of plant materials with solely its endogenous enzymes (Kaoutari et al., 2013). For detritivorous invertebrates, symbiont‐mediated CAZyme production appears a common strategy for acquiring sufficient nutrients from their complex dietary repertoire (Bredon et al., 2018; Colman et al., 2012; Warnecke et al., 2007). In terrestrial ecosystems, termites, millipedes and terrestrial isopods are known important decomposers with complementary enzymatic functions provided by their microbiome (Joly et al., 2015; Ni & Tokuda, 2013; Zimmer, 2002).

Isopods, originating from marine environments and successfully colonizing the aquatic and terrestrial environments, represent a potentially excellent study system for examining the role of microbiome in facilitating a detritivorous diet (Bouchon et al., 2016; Bredon et al., 2018, 2019; Lafuente et al., 2021; Zimmer, 2002; Zimmer & Topp, 1998). Even though the discovery of endogenous cellulolytic enzymes in marine and terrestrial isopods (King et al., 2010; Kostanjšek et al., 2010) confounds the precise role of the isopod‐associated microbiome, metagenomic studies have shown that the microbiota found in the digestive system, especially in the hindgut, of terrestrial and aquatic isopods are closely linked to the CAZymes production (Bredon et al., 2018, 2020). The complexity of the bacterial communities associated with the isopod digestive system, especially in the caeca, further suggests a functional role of the microbiome. Two potential nutritional bacteria *Candidatus Hepatoplasma* crinochetorum and *Candidatus Hepatincola* porcellionum were often found in various terrestrial isopod species but not in marine ones (Fraune & Zimmer, 2008; Kostanjšek et al., 2002, 2004; Wang, Stingl, Anton‐Erxleben, Geisler, et al., 2004; Wang, Stingl, Anton‐Erxleben, Zimmer, et al., 2004). Moreover, *Ca. Hepatoplasma* was suggested to increase its host survival on a cellulosic low‐quality diet (Fraune & Zimmer, 2008), and the recently published genome of this bacterium indicates its potential in producing lignocellulose‐degrading enzymes (Bredon et al., 2020; Leclercq et al., 2014). Wang et al. (2007) showed the presence of four proteobacterial genera *Rhodobacter*, *Burkholderia*, Aeromonas, and *Rickettsiella* in the caeca of freshwater isopods. Except *Rickettsiella*, these bacteria are putatively associated with degradation of plant polymers, phenolic compounds or polycyclic aromatic hydrocarbons. In addition to certain vertically acquired bacteria like *Wolbachia* (Bouchon et al., 2008) and *Rickettsiella* (Cordaux et al., 2007), a considerable fraction of the bacterial diversity in isopod digestive system derives from the environment (Dittmer et al., 2016; Horváthová et al., 2016). Hindgut and caeca form different microhabitats for the environmentally acquired microbes (Dittmer et al., 2016). Given the different roles of these two organs in digestion, proliferation of the beneficial microbial communities, if any, should support their corresponding functions and many of these host‐associated enriched microbes should be key players in the decomposition of complex organic matter, including lignocellulose.

The interaction between isopods and environmental microbes is complex, and extensive studies on terrestrial isopods have shown that diet significantly influences the diversity and abundance of the gut microbiota, and host‐associated microbiota and environmental microbes largely overlap (Dittmer et al., 2016). Thus, consolidating the role of the gut microbiome in nutrition and digestion in isopods requires further individual‐level studies in natural habitats. This is because available microorganisms differ significantly across habitats, and environmental microbes within the digestive system can be transient passengers acting as food resources (Horváthová et al., 2015; Ihnen & Zimmer, 2008), or become stably associated with a host. Individual‐level robustness of such associations is a likely signature of continued enzymatic activities for processing food (Hassall & Jennings, 1975; Horváthová et al., 2016; Horváthová & Bauchinger, 2019). However, the impacts of the resident microbes can also be pathogenic or beneficial in other aspects. Individual‐level microbiome studies in different habitats are therefore important for understanding whether the hypothesized convergent selection on the microbiota with lignocellulose degradation functions exists, and varies, across different individuals in different habitats.

**FIGURE 1:**
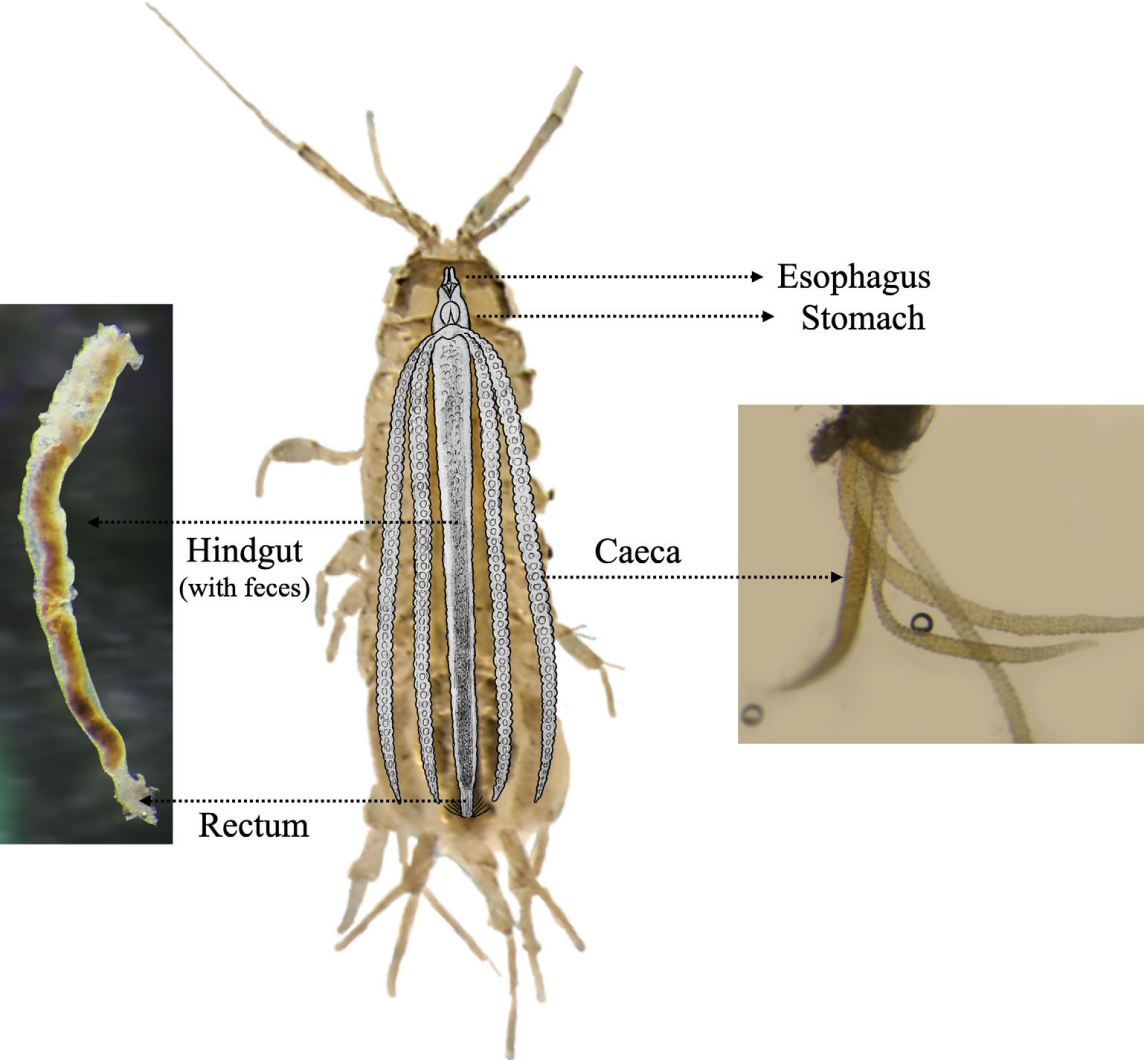
Digestive system of the *Asellus aquaticus* (*Source*: adapted from Bronn et al., 1859).Ingested food is mechanically processed in the foregut (oesophagus and stomach). The majority of food processing takes place in the hindgut, and the caeca are responsible for both digestion and absorption. These tube‐like glands secrete digestive fluids to the stomach and process liquids, as well as fine particles from the hindgut.

The isopod *Asellus aquaticus* (Linnaeus 1758) (Asellota: Asellidae) is one of the most common freshwater detritivorous crustaceans and is widely distributed throughout Europe (Sket, 1994; Verovnik et al., 2005; for review, see Lafuente et al., 2021). Aquatic invertebrates such as *A. aquaticus* (Reiss et al., 2010), but also many *Gammarus* spp. (Nelson, 2011), contribute significantly to the decomposition and cycling of energy and organic matter. However, unlike its terrestrial relatives though having a similar digestive system (Figure 1; Bronn et al., 1859), *A. aquaticus* can only digest these plant detritus with enzymes provided by hosted microbes or ingested enzymes along with the food (Zimmer & Bartholmé, 2003) and the microbiota produces more CAZymes to degrade plant polysaccharides than the host itself (Bredon et al., 2020). Previous studies into the *A. aquaticus* microbiome have either been restricted by experiment methods (low microbial diversity detected) or conducted on pooled samples, obscuring the digestive system enzymatic and microbiota profiles at the individual level.

This study used 16S rRNA amplicon sequencing to characterize the diversity and abundance of microbiota in different digestive organs of the aquatic isopod species, *Asellus aquaticus*, originating from two contrasting freshwater environments, a lake and a stream. The hindgut and the caeca were dissected and collected separately for each individual and to investigate the relationship between host‐associated microbiota and environmental microbes, faecal samples were also collected for each individual. Individual‐level analyses were conducted to assess (i) the tissue‐specific and habitat‐specific diversity and composition of the bacterial communities, (ii) the presence and abundance of possible mutualistic symbionts in hindgut and caeca, and (iii) the individual‐level variation of the host‐associated microbiota within and across habitats.

## RESULTS AND DISCUSSION

We reconstructed the microbiome with zero‐radius OTUs (ZOTUs or amplicon sequence variant [ASV]) with an additional 97% clustering (see Supplementary Information S1 more details). After rarefaction, 2465 ZOTUs assigned to 29 phyla, 78 classes, 130 orders, 228 families and 338 genera remained in the sample dataset. Diversity of bacteria found in the collective digestive system of *A. aquaticus* was notably high (>300 genera). Moreover, the microbiome of the studied *A. aquaticus* is more diverse than that of the terrestrial isopods at all taxonomic levels (Bouchon et al., 2016; Bredon et al., 2018; Dittmer et al., 2016; Horváthová et al., 2016). For example, while microbiome of the terrestrial isopods is predominantly occupied by Proteobacteria (approximately 85%) at the phylum level, the freshwater isopod microbiome consists of approximately 58% Proteobacteria, 27% Bacteroidetes, 10% Tenericutes, 1% Actinobacteria, 1% Firmicutes, 1% Cyanobacteria and 1% other phyla.

**FIGURE 2:**
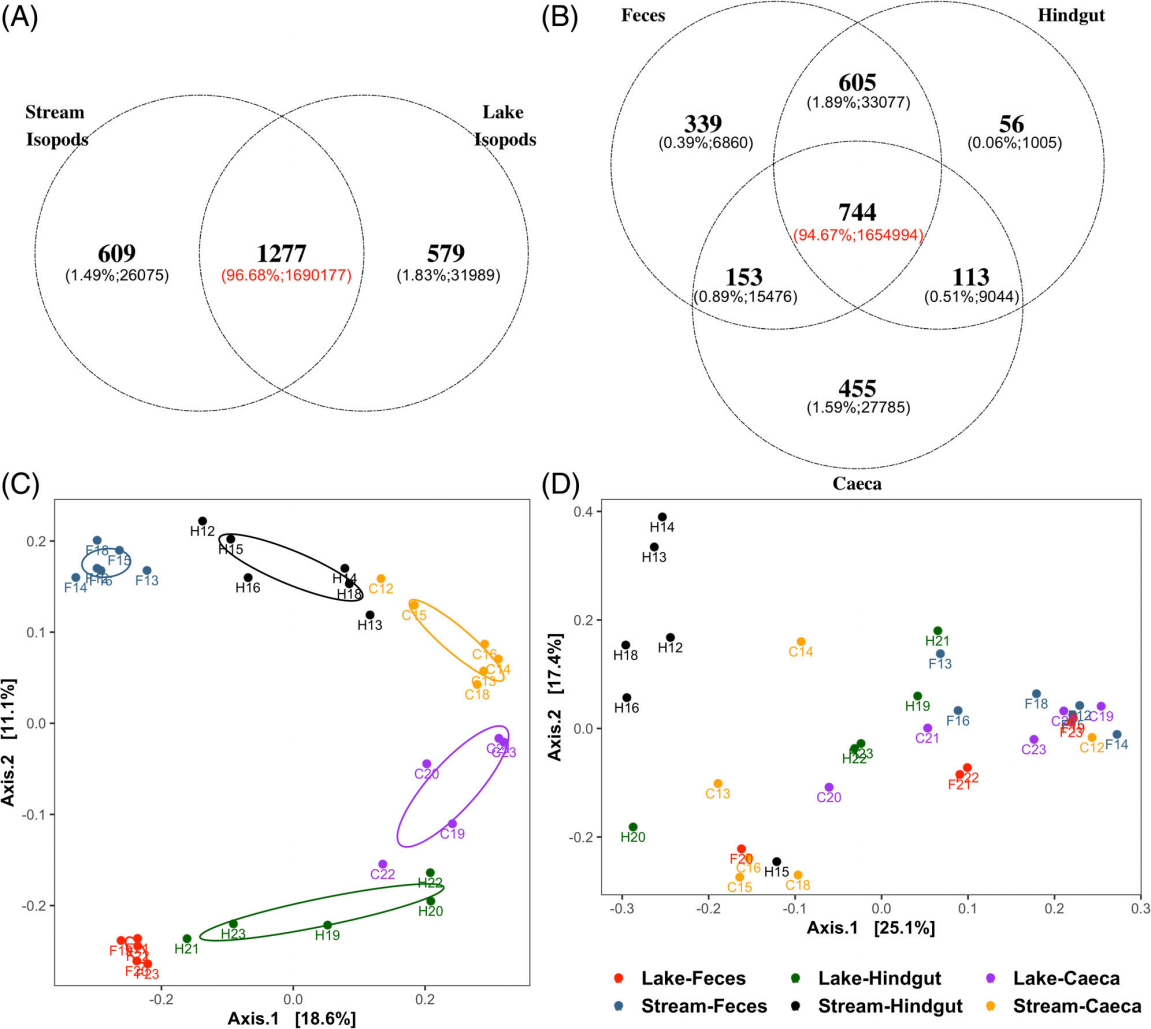
(A) ZOTU distribution and the corresponding abundance in lake and stream individuals. (B) ZOTU distribution and the corresponding abundance in caeca, hindguts, and faeces samples. Within the bracket shows the percentage of the overall abundance and the corresponding abundance. (C) PCoA based on unweighted UniFrac distance matrix showing differences in bacterial community composition between different habitats in different sample types. (D) PCoA based on weighted UniFrac distance matrix showing differences in bacterial community composition among samples from different individuals. Each dot represents a sample, and the number label shows which individual the sample is from. C, caeca; F, faeces; H, hindgut

Individuals from the lake and stream shared 50% of the bacterial ZOTUs, which accounted for 97% of the total bacterial abundance (Figure 2A); 744 ZOTUs which comprised 94.67% of the total abundance were shared by the faeces, caeca and hindgut (Figure 2B). Nonetheless, ZOTU richness (Chao1) and Shannon Diversity varied in the three sample types from lake and stream individuals (Figure S2.1, Table S3.1). We identified the host habitat as the most important factor associated with the difference in microbiota composition (Figure S2.4). Dissimilarity of the tissue‐specific bacterial communities from the lake and stream individuals was highest in the faecal samples, intermediate in the hindgut and became least dissimilar in the caeca (PCoA based on unweighted UniFrac distance matrix, Figure 2C). The dominant source of microbial communities observed in the faecal samples presumably is the environment and the bacteria consumed with plant materials. Although most of the bacterial taxa were shared between the two habitats, the composition of the faecal microbiomes still demonstrates a strong habitat‐specific pattern, pointing to significantly different assemblage of prevailing bacteria in different habitats. PCoA based on weighted UniFrac distance matrix showed that the three sample types clustered differently for each individual (Figure 2D). The isopods in this study were starved for 24 h before the digestive organs were sampled, to isolate most of the ingested microbiota in the faecal samples and to reveal the stable, or resident, part of the host gut microbiota. The faecal and hindgut samples clustered separately in ordination analyses, showing that starvation was effective in most cases in separating the more stable gut communities from the ingested bacteria. The overlap between the digestive organ microbiome and the faecal microbiome among the lake and the river habitat, and the variation among individuals within habitat strongly suggests that the host environment shapes the community composition of the microbes found in the digestive system, especially in the hindguts. Consumption of biofilms along with plant tissues by *A. aquaticus* may explain the observed habitat‐associated variation in microbial community structure in the digestive tissues, and is supported by studies on other detritivorous invertebrates (Bredon et al., 2018; Colman et al., 2012; Dittmer et al., 2016). Patterns seen in the PCoA plots are supported by a significant interaction of Tissue and Habitat reported in the permutational multivariate ANOVA (PERMANOVA) analysis based on unweighted and weighted UniFrac distance matrix (Table 1). PCoA and the associated PERMANOVA statistics showed similar patterns when only common ZOTUs (*n* = 744) shared among the three sample types were included (Figure S2.2, Figure S2.3; Table S3.2, Table S3.3).

**TABLE 1:** Results of permutational multivariate analysis of variance (adonis function)

		Df	Sums Sqs	Mean Sqs	F.Model	*R* ^2^	Pr (>F)
**Unweighted** UniFrac Distance Matrix	Habitat	1	0.8867	0.88673	4.2699	0.0985	0.00001***
	Tissue	2	1.8353	0.91763	4.4186	0.2039	0.00001***
	Habitat:Tissue	2	0.6707	0.33536	1.6148	0.0745	0.01299*
	Residuals	27	5.6072	0.20767		0.623	
	Total	32	8.9999			1	
**Weighted** UniFrac Distance Matrix	Habitat	1	0.3452	0.34519	3.3659	0.0756	0.00108**
	Tissue	2	0.9984	0.49921	4.8678	0.2188	0.00001***
	Habitat:Tissue	2	0.4511	0.22555	2.1993	0.0988	0.00389**
	Residuals	27	2.769	0.10255		0.6067	
	Total	32	4.5637			1	

*Note*: Test is based on either **unweighted** or **weighted** UniFrac distances and 99,999 permutations.

* *P* ≤ 0.05.

** *P* ≤ 0.01.

*** *P* ≤ 0.001.

**FIGURE 3:**
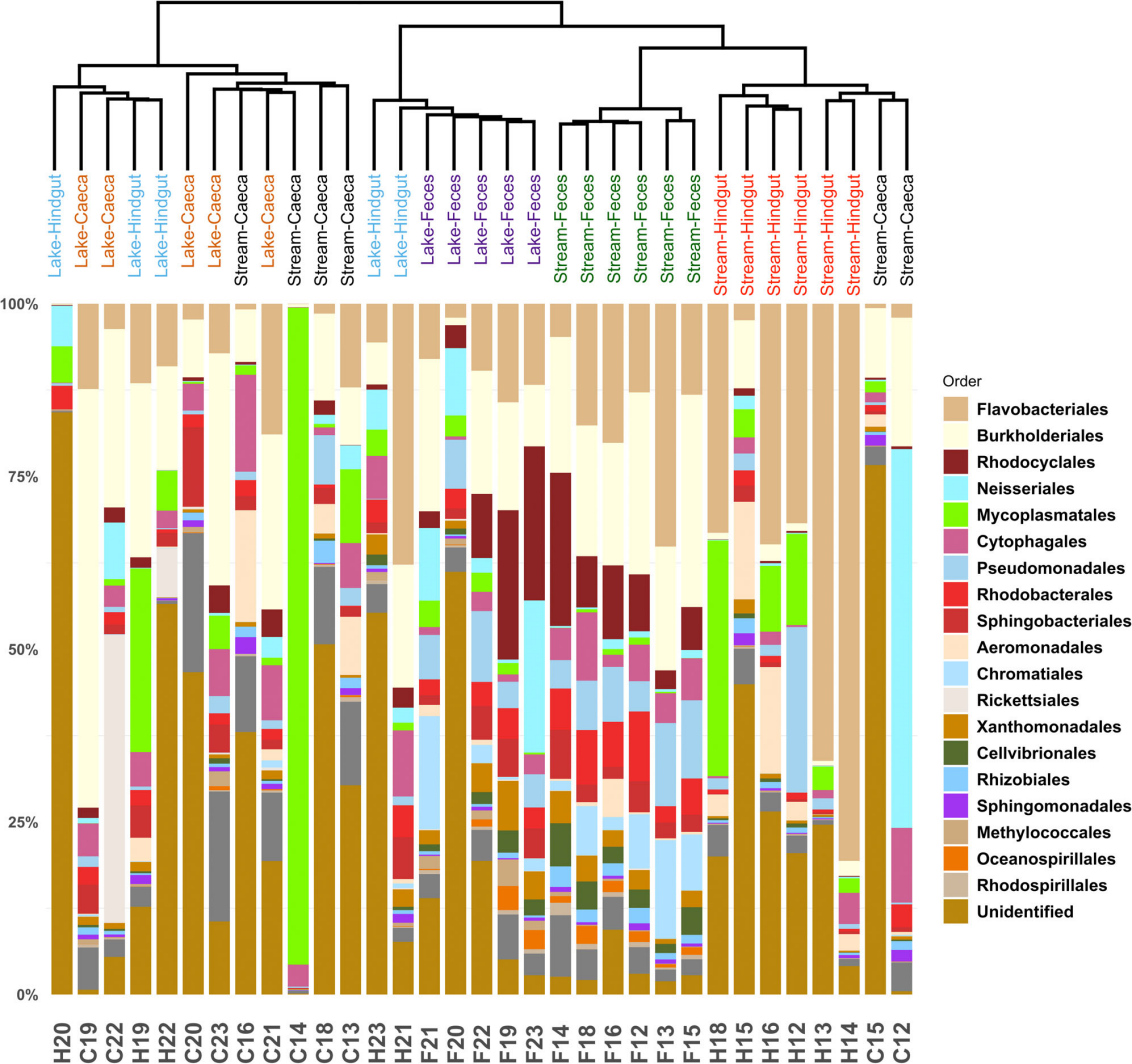
Composition of bacterial communities (relative abundance) in caeca and hindguts. The order of taxa in the legend follows the order of taxa in the community bar (from top to bottom). The 20 most abundant bacterial orders are specified in the legend. Samples are clustered based on unweighted UniFrac distance matrix. C, caeca; F, faeces; H, hindgut

**FIGURE 4:**
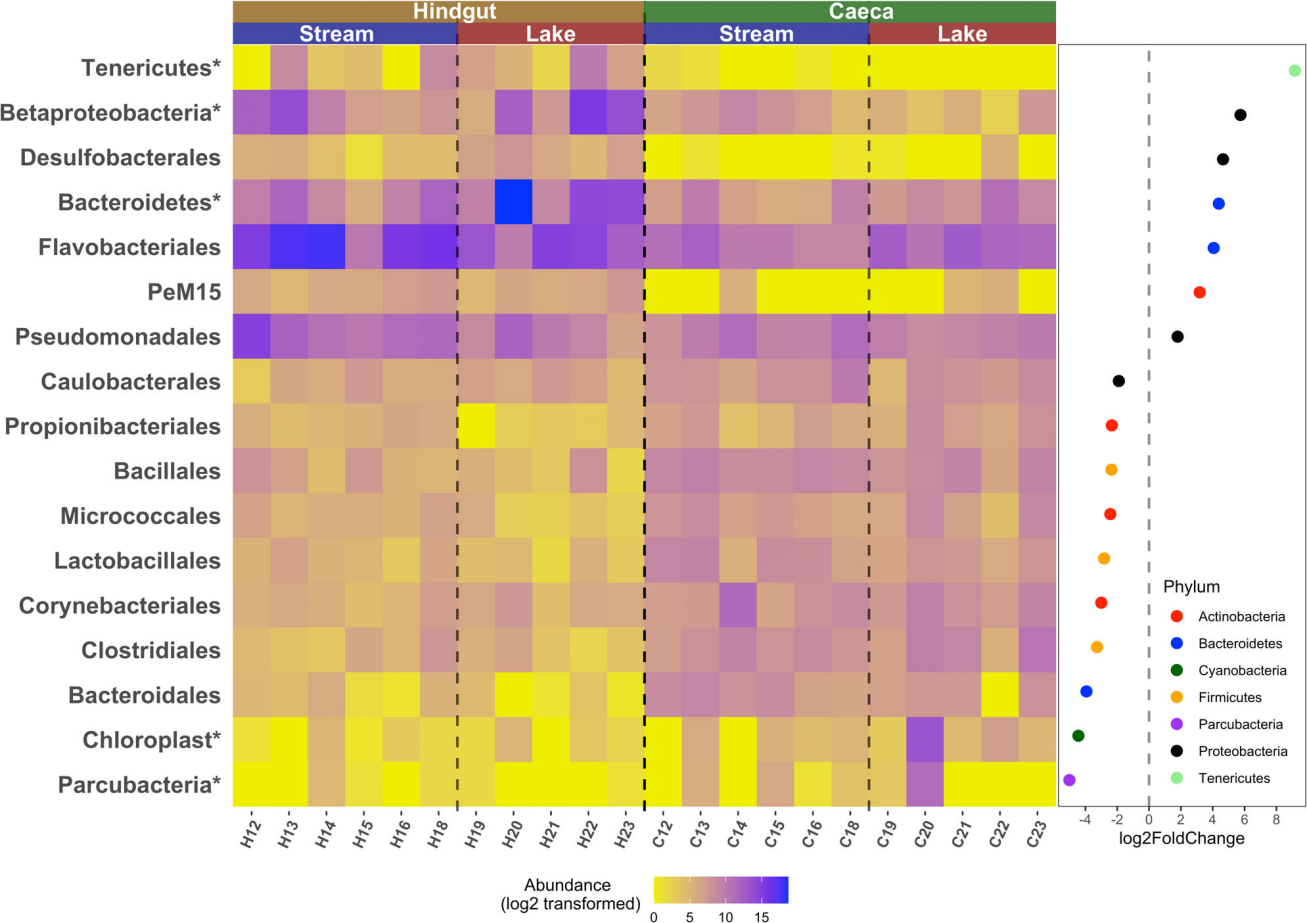
Normalized counts of differentially enriched bacterial orders in hindgut and caeca and the corresponding log2FoldChange (hindgut vs. caeca). Only bacterial orders with adjusted *p* value <0.05 and estimated base mean > 30 are considered as differentially enriched taxa. Unidentified taxa at order level are marked with asterisk. C, caeca; H, hindgut

Bacterial composition in hindguts differed significantly from the composition in caeca both on the individual and on the population (habitat) levels (Figure 3, composition plotted at order level). Both caeca and hindguts shared many abundant Proteobacteria and Tenericutes (assessed by the DESeq2 calculated base mean value, *p*adj >0.05). Bacteroidetes were highly abundant in hindgut samples (DESeq2, base mean = 43,595, log2FoldChange = 2.51, *p*adj <0.001), while caeca microbiome comprised more abundance of Actinobacteria (DESeq2, base mean = 973, log2FoldChange = 1.48, *p*adj <0.05) and Firmicutes (DESeq2, base mean = 758, log2FoldChange = 3.36, *p*adj <0.001). The 10 most abundant orders in the host tissues (hindgut and ceaca) were Flavobacteriales, Burkholderiales, Mycoplasmatales, Cytophagales, Neisseriales, Aeromonadales, Pseudomonadales, Rickettsiales, Sphingobacteriales and Rhodobacterales (Figure 3). These highly predominant bacteria (accounting for 61% of the bacterial abundance in the host tissues) may constitute towards the potential host‐associated core microbiome. DESeq2 analysis indicated that 17 bacterial orders were differentially enriched in either hindguts or caeca (Figure 4). The orders Flavobacteriales, Pseudomonadales, Desulfobacterales and PeM15 were more abundant in the hindgut microbiome. Pseudomonadales are considered the primary symbiont group in many terrestrial isopod populations (Bredon et al., 2019; Dittmer et al., 2016; Dittmer & Bouchon, 2018; Zimmer, 2002). Flavobacteriales was indicated as one of the largest contributors to lignocellulose‐degrading CAZymes in *A. aquaticus* populations (Bredon et al., 2020) and were found in high abundance in each individual in this study. Desulfobacterales is a group of anaerobic bacteria often found in termites and cockroaches and implicated in lignocellulose digestion (Ni & Tokuda, 2013). These bacteria require complex morphological structures in the hindgut to proliferate (Kostanjšek et al., 2004), suggesting that their consistent detection, albeit at low abundances, may indicate their role in the resident functional microbiome, rather than transient passengers. Other highly abundant bacteria like Mycoplasmatales are often found in termite hindguts, where they are shown to participate in depolymerizing cellulose and hemicellulose (Yang et al., 2005). These differentially enriched bacteria and predominant bacteria are prime candidates in support of the robust digestive functions in the hindgut.

While the hindgut microbiome was relatively consistent in its composition across different individuals in the two habitats, the variation in the caeca microbiome among individuals from the same habitat, as well as the variation between habitats, was large (Figures 3 and 4). Although Burkholderiales, Neisseriales, Mycoplasmatales and Rickettsiales species were highly abundant in some caeca samples, they were not found in every individual. Micrococcales and Burkholderiales are known to contribute significantly to polymer degradation (Bredon et al., 2020), but the individual level variation in their dominance and abundance between caeca samples suggests that the significance of their role in digestion is unclear. Recently, it was shown that it is hindgut microbiome rather than caeca microbiome that contributes significantly to the lignocellulose degradation (Bredon et al., 2020). Our results support this view, as no specific bacteria in the caeca was found as “obligate” for the host and to be known to participate in cellulose digestion. Nonetheless, composition of the caeca microbiome was more stable and convergent across the two habitats (less variance explained by habitat) than the hindgut and faecal samples (Figure 2C). Meanwhile, caeca harboured the largest number of tissue‐specific ZOTUs (*n* = 455, only detected in a given tissue type), although these unique ZOTUs were of low abundance (Figure 2B). Piphillin metagenome reconstruction and prediction of the functional profile of the bacterial communities implied that the caeca microbiome was richer in genes that are involved in lipid metabolism, glycan biosynthesis and metabolism, and other digestive system‐related pathways, compared to the metagenome inferred from hindguts (Table 2). The analysis does indirectly hint at an accessory role for the caeca microbiome functions, relative to the hindgut; however, the functional profile prediction must be interpreted with caution, as only 368 KEGG pathways were assigned.

**TABLE 2:** KEGG pathways differentially represented in hindguts and caeca

Pathway	Caeca	Hindgut	Related biological process
ko00062	7.69 ± 1.54	4.96 ± 1.81	Lipid metabolism*
ko00571	11.71 ± 0.97	9.86 ± 1.13	Glycan biosynthesis and metabolism**
ko03050	8.97 ± 1.56	6.80 ± 1.28	Folding, sorting and degradation**
ko04916	8.41 ± 1.73	5.13 ± 1.53	Endocrine system***

*Note*: Abundance is log2 transformed. Asterisks indicate the range of adjusted *p* values: *padj ≤ 0.05; **padj ≤ 0.01; ***padj ≤ 0.001.

Caeca, though not having a consistent microbiome profile, were often associated with taxa implicated in endosymbiotic/parasitic roles. Notably, *Wolbachia* (order Rickettsiales) was more abundant in lake caeca, while *Ca*. *Hepatoplasma* (order Mycoplasmatales) was more enriched in stream caeca (Table S3.4). Two individuals carried relatively more *Ca*. *Hepatoplasma* in caeca (95.1% and 4.3% of the caeca microbiomes, C14 and C23 in Figure 3 respectively) and one individual was infected by *Wolbachia* (order Rickettsiales), which dominated the microbiome of this individual (41.1% of caeca microbiome, C22 in Figure 3). In total, *Wolbachia* was present (>50 reads after rarefaction) in samples from three individuals (C22, H22, C23, F12), *Ca*. *Hepatoplasma* from three individuals (C14, C22, C23) and *Ca*. *Hepatincola* (order Rickettsiales) from three individuals (F19, F21, C23) (Table S3.5).

It is suggested that *Ca*. *Hepatoplasma* is environmentally transmitted (Fraune & Zimmer, 2008), but no, or few *Ca*. *Hepatoplasma* (0 or <50 reads) were found in the faecal samples in this study. The near‐absence from faeces microbiome may reflect ecological constrains, or that *Ca*. *Hepatoplasma* environmental transmission may occur more readily in high‐density populations or during juvenile stages. To confirm on the transmission routes, further experimental work will be required. Detection of both *Ca. Hepatoplasma* and *Ca. Hepatincola* in individual 23 in our study (>50 reads after rarefaction) suggests higher lability of the *Asellus* microbiome as no individual in the previous studies on terrestrial isopods were found infected with both bacteria simultaneously (Bouchon et al., 2016; Fraune & Zimmer, 2008; Wang et al., 2007). Considering the widespread occurrence of these bacteria, and their monopoly in the glands of some isopods, further functional investigations based on metatranscriptomics will be necessary to confirm and validate the specific functional contributions of these bacteria in the caeca if there are any.

Structuring the microbiome profile on the individual level also allows us to identify individuals co‐infected by both *Ca. Hepatoplasma* and *Wolbachia* (individual 22, 23; >50 reads after rarefaction). *Wolbachia* are facultative intracellular symbionts (Zug & Hammerstein, 2012), Terrestrial isopod populations are mostly infected by the vertically acquired *Wolbachia* B group (inducing feminization or cytoplasmic incompatibilities), and its presence influences the microbial communities in the isopod populations (Bouchon et al., 1998; Dittmer & Bouchon, 2018). While the presence of *Ca. Hepatoplasma* promotes host survival on lignocellulose‐rich diet, the nutritional role of *Wolbachia* and its interactions with other endosymbionts are unknown. Further studies focused on measuring the prevalence of *Wolbachia* in larger sample sizes and diverse *A. aquaticus* populations may be interesting for understanding its role in mediating eco‐evolutionary feedbacks between the holobiont and isopod host (Lafuente et al., 2021).

Our study provides the first individual level isopod microbiome data, which will enable further studies on the interactions between the host, parasites, and the microbiome. The *A. aquaticus* caeca and hindgut microbiomes at individual level have shown both flexibility and consistency, which underlies the importance of a functional core microbiome in a wide range of detritivorous isopods. Our study further confirms that caeca and hindgut form two different microhabitats for bacteria and contain tissue‐specific microbial communities. While predominant and differentially enriched taxa in the hindgut support the robust digestive function, a more diverse microbiome is found in caeca aligning with the miscellaneous functions of the organ.

A relatively stable caeca microbiome was detected, suggesting that selective enrichment system may exist in the caeca to generate a specific microbiome composition and functionality. The detection of *Wolbachia* and *Ca. Hepatoplasma* reveals a more complicated interaction between the host and the microbial communities in the caeca than between the host and the hindgut microbiome. Questions regarding the role of the microbiome in facilitating the spread of the isopod populations, enabling the high polymer degradation activity, and helping the host to cope with different stressors remain to be answered. The complexity and variation of the *A. aquaticus* microbiome on the individual level uncovered in this study further emphasizes freshwater isopods as excellent model organism for studying the ecology and evolution of host–microbiome interactions (Lafuente et al., 2021; O'Callaghan et al., 2019).

## AUTHOR CONTRIBUTIONS

**Aijuan Liao:** Conceptualization, lead of Investigation, Methodology, Data curation, Formal analysis, Figures preparation, and Writing‐original draft; **Hanna Hartikainen** and **Claudia C. Buser:** Conceptualization, supportingof Investigation, Data analysis, Writing‐original draft, and lead of Supervision and Review & Editing.

## CONFLICT OF INTEREST

The authors have no conflict of interest to declare.

## Supporting information


**Data S1:** supporting information

## Data Availability

All raw reads can be accessed via the Sequence Read Archive (SRA archive): Accession Number PRJNA884879 (https://www.ncbi.nlm.nih.gov/bioproject/PRJNA884879).

## References

[emi413142-bib-0001] Bouchon, D. , Cordaux, R. & Grève, P. (2008) Feminizing *Wolbachia* and the evolution of sex determination in isopods. In: Insect symbiosis, Vol. 3. FL: CRC Press, pp. 273–294.

[emi413142-bib-0002] Bouchon, D. , Rigaud, T. & Juchault, P. (1998) Evidence for widespread *Wolbachia* infection in isopod crustaceans: molecular identification and host feminization. Proceedings of the Royal Society of London ‐ Series B: Biological Sciences, 265, 1081–1090. 10.1098/rspb.1998.0402 PMC1689171 9684374

[emi413142-bib-0003] Bouchon, D. , Zimmer, M. & Dittmer, J. (2016) The terrestrial isopod microbiome: an all‐in‐one toolbox for animal–microbe interactions of ecological relevance. Frontiers in Microbiology, 7, 1472. 27721806 10.3389/fmicb.2016.01472 PMC5033963

[emi413142-bib-0004] Bredon, M. , Dittmer, J. , Noël, C. , Moumen, B. & Bouchon, D. (2018) Lignocellulose degradation at the holobiont level: teamwork in a keystone soil invertebrate. Microbiome, 6, 162. 30223906 10.1186/s40168-018-0536-y PMC6142342

[emi413142-bib-0005] Bredon, M. , Herran, B. , Bertaux, J. , Grève, P. , Moumen, B. & Bouchon, D. (2020) Isopod holobionts as promising models for lignocellulose degradation. Biotechnology for Biofuels, 13, 49. 32190114 10.1186/s13068-020-01683-2 PMC7071664

[emi413142-bib-0006] Bredon, M. , Herran, B. , Lheraud, B. , Bertaux, J. , Grève, P. , Moumen, B. et al. (2019) Lignocellulose degradation in isopods: new insights into the adaptation to terrestrial life. BMC Genomics, 20, 462. 31174468 10.1186/s12864-019-5825-8 PMC6555040

[emi413142-bib-0007] Bronn, H.G. , Gerstaecker, A. , Hoffmann, C.K. , Keferstein, W.M. & Ortmann, A.E. (1859) Arthropoden. In: Die Klassen und Ordnungen des Thier‐Reichs, wissenschaftlich dargestellt in Wort und Bild. C.F. Winter: Leipzig und Heidelberg, pp. 8–278.

[emi413142-bib-0008] Colman, D.R. , Toolson, E.C. & Takacs‐Vesbach, C.D. (2012) Do diet and taxonomy influence insect gut bacterial communities? Molecular Ecology, 21, 5124–5137. 22978555 10.1111/j.1365-294X.2012.05752.x

[emi413142-bib-0009] Cordaux, R. , Paces‐Fessy, M. , Raimond, M. , Michel‐Salzat, A. , Zimmer, M. & Bouchon, D. (2007) Molecular characterization and evolution of arthropod‐pathogenic *Rickettsiella* bacteria. Applied and Environmental Microbiology, 73, 5045–5047. 17557851 10.1128/AEM.00378-07 PMC1951046

[emi413142-bib-0010] Dittmer, J. & Bouchon, D. (2018) Feminizing *Wolbachia* influence microbiota composition in the terrestrial isopod *Armadillidium vulgare* . Scientific Reports, 8, 6998. 29725059 10.1038/s41598-018-25450-4 PMC5934373

[emi413142-bib-0011] Dittmer, J. , Lesobre, J. , Moumen, B. & Bouchon, D. (2016) Host origin and tissue microhabitat shaping the microbiota of the terrestrial isopod *Armadillidium vulgare* . FEMS Microbiology Ecology, 92, fiw063. 27004796 10.1093/femsec/fiw063

[emi413142-bib-0012] Fenoy, E. , Moyano, F.J. & Casas, J.J. (2020) Warming and nutrient‐depleted food: two difficult challenges faced simultaneously by an aquatic shredder. Freshwater Science, 39, 393–404.

[emi413142-bib-0013] Fraune, S. & Zimmer, M. (2008) Host‐specificity of environmentally transmitted mycoplasma‐like isopod symbionts. Environmental Microbiology, 10, 2497–2504. 18833647 10.1111/j.1462-2920.2008.01672.x

[emi413142-bib-0014] Hassall, M. & Jennings, J.B. (1975) Adaptive features of gut structure and digestive physiology in the terrestrial isopod *Philoscia muscorum* (scopoli) 1763. The Biological Bulletin, 149, 348–364. 1203332 10.2307/1540531

[emi413142-bib-0015] Horváthová, T. , Babik, W. & Bauchinger, U. (2016) Biofilm feeding: microbial colonization of food promotes the growth of a detritivorous arthropod. ZooKeys, 577, 25–41. 10.3897/zookeys.577.6149 PMC4829882 27110187

[emi413142-bib-0016] Horváthová, T. & Bauchinger, U. (2019) Biofilm improves isopod growth independent of the dietary cellulose content. Physiological and Biochemical Zoology, 92, 531–543. 31556843 10.1086/705441

[emi413142-bib-0017] Horváthová, T. , Kozłowski, J. & Bauchinger, U. (2015) Growth rate and survival of terrestrial isopods is related to possibility to acquire symbionts. European Journal of Soil Biology, 69, 52–56.

[emi413142-bib-0018] Ihnen, K. & Zimmer, M. (2008) Selective consumption and digestion of litter microbes by *Porcellio scaber* (isopoda: Oniscidea). Pedobiologia, 51, 335–342.

[emi413142-bib-0019] Joly, F.‐X. , Coulis, M. , Gérard, A. , Fromin, N. & Hättenschwiler, S. (2015) Litter‐type specific microbial responses to the transformation of leaf litter into millipede feces. Soil Biology and Biochemistry, 86, 17–23.

[emi413142-bib-0020] Kaoutari, A.E. , Armougom, F. , Gordon, J.I. , Raoult, D. & Henrissat, B. (2013) The abundance and variety of carbohydrate‐active enzymes in the human gut microbiota. Nature Reviews. Microbiology, 11, 497–504. 23748339 10.1038/nrmicro3050

[emi413142-bib-0021] King, A.J. , Cragg, S.M. , Li, Y. , Dymond, J. , Guille, M.J. , Bowles, D.J. et al. (2010) Molecular insight into lignocellulose digestion by a marine isopod in the absence of gut microbes. Proceedings of the National Academy of Sciences, 107, 5345–5350. 10.1073/pnas.0914228107 PMC2851747 20212162

[emi413142-bib-0022] Kostanjšek, R. , Lapanje, A. , Rupnik, M. , Štrus, J. , Drobne, D. & Avguštin, G. (2004) Anaerobic bacteria in the gut of terrestrial isopod crustacean *Porcellio scaber* . Folia Microbiologia (Praha), 49, 179–182. 15227793 10.1007/BF02931397

[emi413142-bib-0023] Kostanjšek, R. , Milatovič, M. & Štrus, J. (2010) Endogenous origin of endo‐β‐1,4‐glucanase in common woodlouse *Porcellio scaber* (crustacea, isopoda). Journal of Comparative Physiology. B, Biochemical, Systemic, and Environmental Physiology, 180, 1143–1153. 20544203 10.1007/s00360-010-0485-7

[emi413142-bib-0024] Kostanjšek, R. , Štrus, J. & Avguštin, G. (2002) Genetic diversity of bacteria associated with the hindgut of the terrestrial crustacean *Porcellio scaber* (crustacea: isopoda). FEMS Microbiology Ecology, 40, 171–179. 19709225 10.1111/j.1574-6941.2002.tb00950.x

[emi413142-bib-0025] Lafuente, E. , Lürig, M.D. , Rövekamp, M. , Matthews, B. , Buser, C. , Vorburger, C. et al. (2021) Building on 150 years of knowledge: the freshwater isopod *Asellus aquaticus* as an integrative eco‐evolutionary model system. Frontiers in Ecology and Evolution, 9, 699.

[emi413142-bib-0026] Leclercq, S. , Dittmer, J. , Bouchon, D. & Cordaux, R. (2014) Phylogenomics of “Candidatus Hepatoplasma crinochetorum,” a lineage of mollicutes associated with noninsect arthropods. Genome Biology and Evolution, 6, 407–415. 24482531 10.1093/gbe/evu020 PMC3942034

[emi413142-bib-0027] Nelson, D. (2011) *Gammarus*‐microbial interactions: a review. International Journal of Zoology, 2011, e295026.

[emi413142-bib-0028] Ni, J. & Tokuda, G. (2013) Lignocellulose‐degrading enzymes from termites and their symbiotic microbiota. Biotechnology Advances, 31, 838–850. 23623853 10.1016/j.biotechadv.2013.04.005

[emi413142-bib-0029] O'Callaghan, I. , Harrison, S. , Fitzpatrick, D. & Sullivan, T. (2019) The freshwater isopod *Asellus aquaticus* as a model biomonitor of environmental pollution: a review. Chemosphere, 235, 498–509. 31276864 10.1016/j.chemosphere.2019.06.217

[emi413142-bib-0030] Reiss, J. , Bailey, R.A. , Cássio, F. , Woodward, G. & Pascoal, C. (2010) Chapter 4 ‐ assessing the contribution of micro‐organisms and macrofauna to biodiversity–ecosystem functioning relationships in freshwater microcosms. In: Woodward, G. (Ed.) Advances in ecological research. Integrative ecology: from molecules to ecosystems. London, UK: Academic Press, pp. 151–176.

[emi413142-bib-0031] Sket, B. (1994) Distribution of *Asellus aquaticus* (crustacea: isopoda: Asellidae) and its hypogean populations at different geographic scales, with a note on *Proasellus istrianus* . Hydrobiologia, 287, 39–47.

[emi413142-bib-0032] Verovnik, R. , Sket, B. & Trontelj, P. (2005) The colonization of Europe by the freshwater crustacean *Asellus aquaticus* (crustacea: isopoda) proceeded from ancient refugia and was directed by habitat connectivity. Molecular Ecology, 14, 4355–4369. 16313598 10.1111/j.1365-294X.2005.02745.x

[emi413142-bib-0033] Wang, Y. , Brune, A. & Zimmer, M. (2007) Bacterial symbionts in the hepatopancreas of isopods: diversity and environmental transmission. FEMS Microbiology Ecology, 61, 141–152. 17506824 10.1111/j.1574-6941.2007.00329.x

[emi413142-bib-0034] Wang, Y. , Stingl, U. , Anton‐Erxleben, F. , Geisler, S. , Brune, A. & Zimmer, M. (2004) “*Candidatus Hepatoplasm*a crinochetorum,” a new, stalk‐forming lineage of mollicutes colonizing the midgut glands of a terrestrial isopod. Applied and Environmental Microbiology, 70, 6166–6172. 15466563 10.1128/AEM.70.10.6166-6172.2004 PMC522098

[emi413142-bib-0035] Wang, Y. , Stingl, U. , Anton‐Erxleben, F. , Zimmer, M. & Brune, A. (2004) “*Candidatus Hepatincola* porcellionum” gen. Nov., sp. nov., a new, stalk‐forming lineage of Rickettsiales colonizing the midgut glands of a terrestrial isopod. Archives of Microbiology, 181, 299–304. 14770277 10.1007/s00203-004-0655-7

[emi413142-bib-0036] Warnecke, F. , Luginbühl, P. , Ivanova, N. , Ghassemian, M. , Richardson, T.H. , Stege, J.T. et al. (2007) Metagenomic and functional analysis of hindgut microbiota of a wood‐feeding higher termite. Nature, 450, 560–565. 18033299 10.1038/nature06269

[emi413142-bib-0037] Yang, H. , Schmitt‐Wagner, D. , Stingl, U. & Brune, A. (2005) Niche heterogeneity determines bacterial community structure in the termite gut (*Reticulitermes santonensis*). Environmental Microbiology, 7, 916–932. 15946289 10.1111/j.1462-2920.2005.00760.x

[emi413142-bib-0038] Zimmer, M. (2002) Nutrition in terrestrial isopods (isopoda: Oniscidea): an evolutionary‐ecological approach. Biological Reviews, 77, 455–493. 12475050 10.1017/s1464793102005912

[emi413142-bib-0039] Zimmer, M. & Bartholmé, S. (2003) Bacterial endosymbionts in *Asellus aquaticus* (Isopoda) and *Gammarus pulex* (Amphipoda) and their contribution to digestion. Limnology and Oceanography, 48, 2208–2213.

[emi413142-bib-0040] Zimmer, M. & Topp, W. (1998) Microorganisms and cellulose digestion in the gut of the woodlouse *Porcellio scaber* . Journal of Chemical Ecology, 24, 1397–1408.

[emi413142-bib-0041] Zug, R. & Hammerstein, P. (2012) Still a host of hosts for *Wolbachia*: analysis of recent data suggests that 40% of terrestrial arthropod species are infected. PLoS One, 7, e38544. 22685581 10.1371/journal.pone.0038544 PMC3369835

