# Supplementary material for: Individual level microbial communities in the digestive system of the freshwater isopod Asellus aquaticus : Complex, robust and prospective
Source: Environ Microbiol Rep. 2023 Feb 13;15(3):188–96. doi: 10.1111/1758-2229.13142 (PMC10464695; doi:10.1111/1758-2229.13142)
Supplement: Supplementary file 1 — Data S1: supporting information [file EMI4-15-188-s001.docx]

Supplementary Information

SI 1: Materials and Methods

SI 2: Supplementary Figures

SI 3: Supplementary Tables

SI 1 Materials and Methods

**Specimen sampling, processing and experimental design**

*A. aquaticus* females were collected by kick-sampling from Lake Lucern (N = 5) and a side channel of the river Thur (N = 6) in October 2018. In the field, isopods were handled with forceps, shaken in sterile water to clean and starved singly for 24 hours in 50ml Falcon tubes filled with sterile water at 22°C. *A. aquaticus* was surface sterilized with dilute bleach before sedation with a dose of carbonated water. In a clean petri dish filled with sterile water, the digestive tract was dissected out under a Leica M205C stereomicroscope using sterilized forceps. Hindgut and the caeca were then carefully separated, rinsed in sterile water and immediately snap-frozen in liquid nitrogen and stored at -80°C prior to analysis. Feces were collected from the tubes and stored similarly.

**DNA extraction and 16S rRNA gene amplicon sequencing**

DNA was extracted using Qiagen DNeasy Blood & Tissue Kit (QIAGEN N.V, Hilden, Germany) according to the manufacturer’s instructions with an added bead-beating step. DNA was stored at −20 °C. A 444-bp-fragment spanning the variable region V3-V4 of the bacterial 16S rRNA gene was amplified using the universal bacterial primers b341F (5’-CCTACGGGAGGCAGCAG -3’) and 785R (5’- CTACCAGGGTATCTAATCC -3’) (Klindworth *et al.* 2013). Both primers were adapted for Illumina MiSeq amplicon sequencing library preparation by adding the Nextera adapter, 0-3 bp random frameshifts and a 19-bp Multiplex Identifier sequence (all primers listed in Table S6). For each sample, three 25 μl PCR reactions were performed and pooled. Each 25 μl PCR reaction contained 1X Qiagen Multiplex PCR MasterMix, 0.3 mM of both forward and reverse primer, and 3 μl of template DNA. PCR cycles were performed as follows: 95 °C for 5 min, followed by 31 cycles of 95 °C for 45 s, 55 °C for 60 s, 72 °C for 60 s and a final extension of 72 °C for 10 min. PCR products were then subjected to a purification with 0.8x self-made SPRI magnetic beads (Genomic Diversity Centre, Zurich). Tailed PCR products were indexed by amplifying for ten cycles with Nextera XT v2 indexing primers using KAPA HiFi HotStart ReadyMix (Roche Holding AG, Basel, Switzerland), followed by another clean-up with self-made SPRI magnetic beads. Each sample was processed separately before normalization and in total, 40 libraries were prepared, including two mock bacteria communities (ZymoBIOMICS Microbial community DNA standard, Zymo Research), two reagent-only negative controls with elute from control DNA extractions (extraction conducted using sterile water instead of tissue sample), as well as three reagent-only controls from the PCR step (sterile water added instead of extracted DNA). Negative and positive controls clustered separately from samples in NMDS ordination based on Bray-Curtis distance (Figure S1.1). Libraries were quantified using Tapestation (Agilent Technologies, Santa Clara, USA) and Qubit (Invitrogen, Carlsbad, USA), normalized and pooled. Pooled libraries were sequenced on the Illumina MiSeq instrument (Illumina Inc., San Diego, USA) using a 600 cycle v3 sequencing kit, paired-end 2 × 300 cycle sequencing mode at the Genetic Diversity Center Zürich (<http://www.gdc.ethz.ch>).

**Data analysis**

Raw reads were quality controlled with FastQC v.0.11.4 (Schmieder and Edwards 2011). Paired reads were merged (FLASH v1.2.9), primers were trimmed with Cutadapt v1.5 (Martin 2011). The joined reads were then quality filtered using PRINSEQ‐lite v0.20.4 (Schmieder and Edwards 2011). Raw fastq files were processed with UNOISE3 and denoised zero-radius OTUs (ZOTUs, also known as amplicon sequence variants (ASVs)) were generated. An additional 97% clustering with cluster_smallmem within the USEARCH workflow (Edgar 2010) was applied in order to create a more stringent error correction. Representative sequences in the filtered dataset were annotated with taxonomic ranks using SINTAX against the SILVA database (Edgar 2016). All further analyses were performed using the Bioconductor library phyloseq 1.30.0 (McMurdie and Holmes 2013), DESeq2 1.26.0 (Love, Huber and Anders 2014), VennDiagram 1.6.20 (Chen and Boutros 2011), vegan 2.5-7 (Oksanen *et al.* 2020) and ggplot2 3.3.3 (Wickham 2016) in the software R 3.6.3 (R Core Team 2020).

ZOTUs classified as Archaea, Mitochondria or Unclassified at Phylum level were excluded, as were ZOTUs represented by fewer than 10 reads in the global dataset (combined abundance < 0.01% of the total raw reads were removed). As reagent and laboratory contamination can critically impact the microbiome analysis for samples with low microbial biomass (Salter *et al.* 2014), ZOTUs with prevalence of more than 0.10% amongst all the negative control bacteria community were regarded as possible contaminants (to increase the strictness of the filtering, negative control with highest abundance was removed before calculating the 0.10% threshold) and therefore, excluded from the sample dataset (resulting in removal of 14.81% of total raw reads and 110 ZOTUs).

Non-metric multidimensional scaling (NMDS) analysis was performed based on Bray-Curtis distances. All samples were rarefied to an equal sampling depth of 52977 reads per sample (sampling without replacement; seed = 20190124) and sampling depth assessed using rarefaction curves (Figure S1.2). Alpha diversity was calculated using Chao1 richness estimator and Shannon Diversity Index. Analysis of variance (ANOVA) with post-hoc Tukey test for multiple comparisons was used to compare alpha diversity among samples grouped by habitat and tissue type. Assumption of non-normality was violated for Shannon Diversity Index, and non-parametric Kruskall-Wallis test, followed by pairwise Wilcoxon signed rank tests were used to confirm the results of the ANOVA. Bacterial community dissimilarity estimates in the caeca, hindgut and feces, and between the habitat types, were tested with permutational multivariate ANOVA (PERMANOVA) using adonis function based on both weighted and unweighted UniFrac distance matrix. Principal Component Analysis (PCoA) was used to visualize the pairwise unweighted and weighted Unirac distance among samples. Venn diagrams were generated with the ‘VennDiagram’ package.

DESeq2 was used to identify differentially enriched taxa and pathways between hindgut and caeca and this analysis was performed on a DESeq normalized dataset. p values attained by Wald test were corrected for multiple testing using the Benjamini-Hochberg method. Only bacterial order with adjusted p values < 0.05 and estimated base mean >30 was considered significantly differentially abundant between two tissues.

**Functional profile prediction**

To investigate the functional profiles of the bacterial communities in hindgut and caeca, we used Piphillin to reconstruct the metagenome with the 16S rRNA sequences (Iwai *et al.* 2016). The analysis was run using a 97% ID cutoff and the predicted genomes were assigned to KEGG pathways with the KEGG database (Kyoto Encyclopedia of Genes and Genomes; release May 2020).


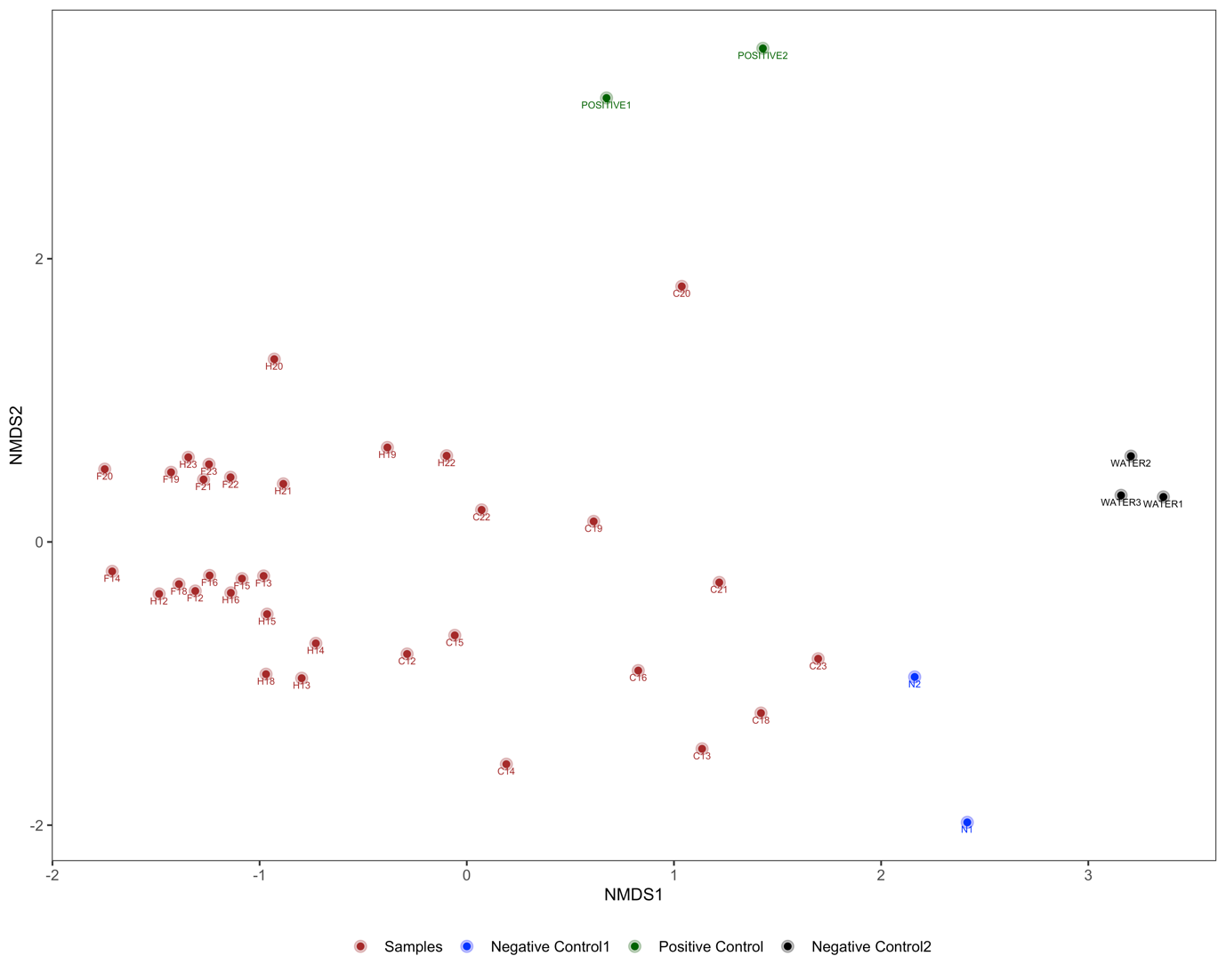


Figure S1.1 Negative and positive controls clustered separately from samples in NMDS ordination based on Bray-Curtis distance (based on unrarefied data). Negative control 1 is empty extracts and negative control 2 is water samples from PCR steps. Positive controls are two mock bacterial communities.


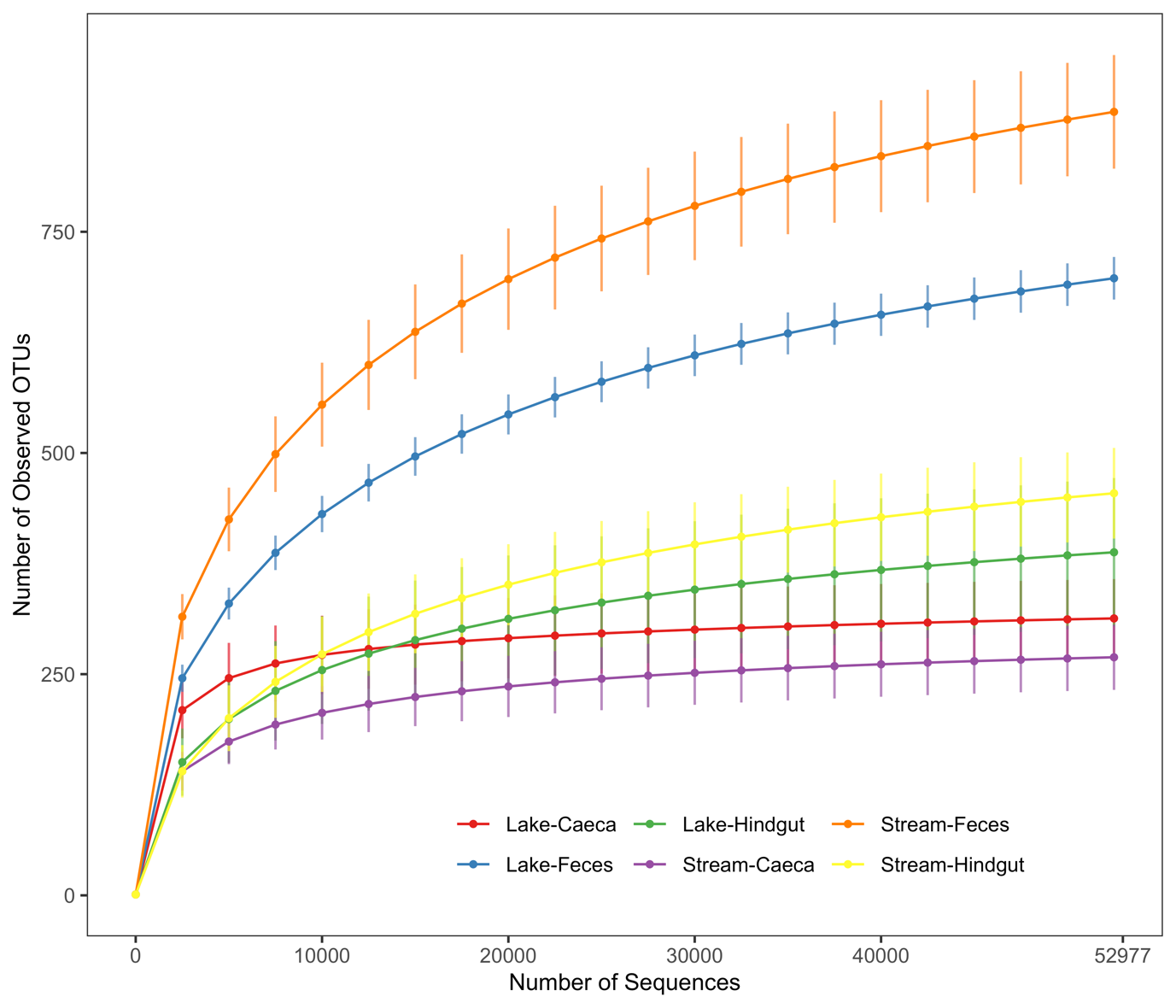


Figure S1.2 Rarefaction curves (mean ± SE) for each sample type. Rarefaction curves indicate high coverage sampling of the bacterial communities of the caeca and hindguts, but the sequencing coverage was not sufficient to exhaustively profile the bacterial communities in the fecal samples.

SI 2 Supplementary Figures


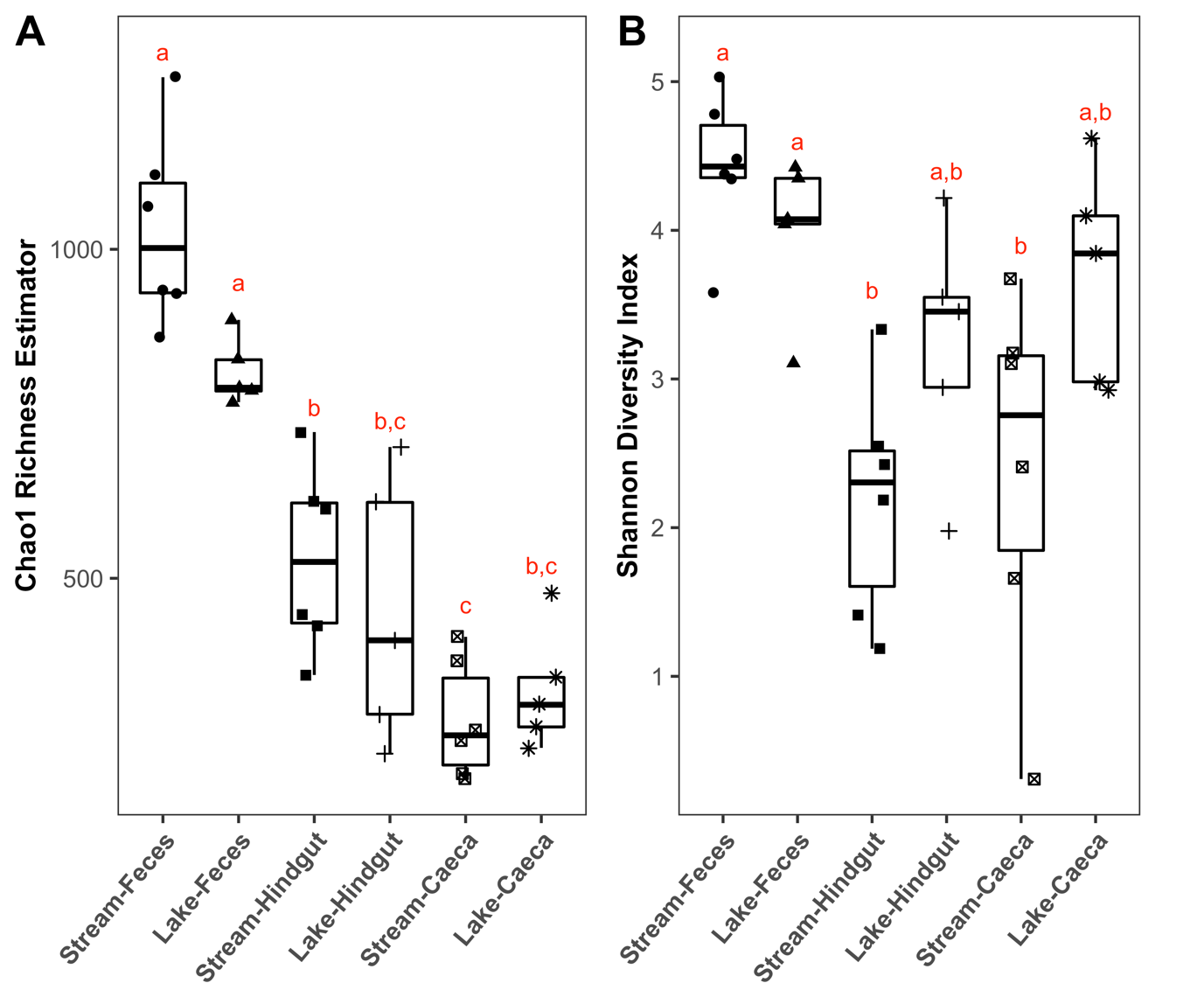


Figure S2.1 **A:** Richness and **B:** diversity indices (mean ± SE). Different letters above each box indicate significant differences acquired from ANOVA with post-hoc Tukey test for multiple comparisons. To validate the ANOVA results, Shannon diversity was further compared to test for significant differences using the non-parametric Kruskal-Wallis test followed by pairwise Wilcoxon signed rank tests (Table S3.1).


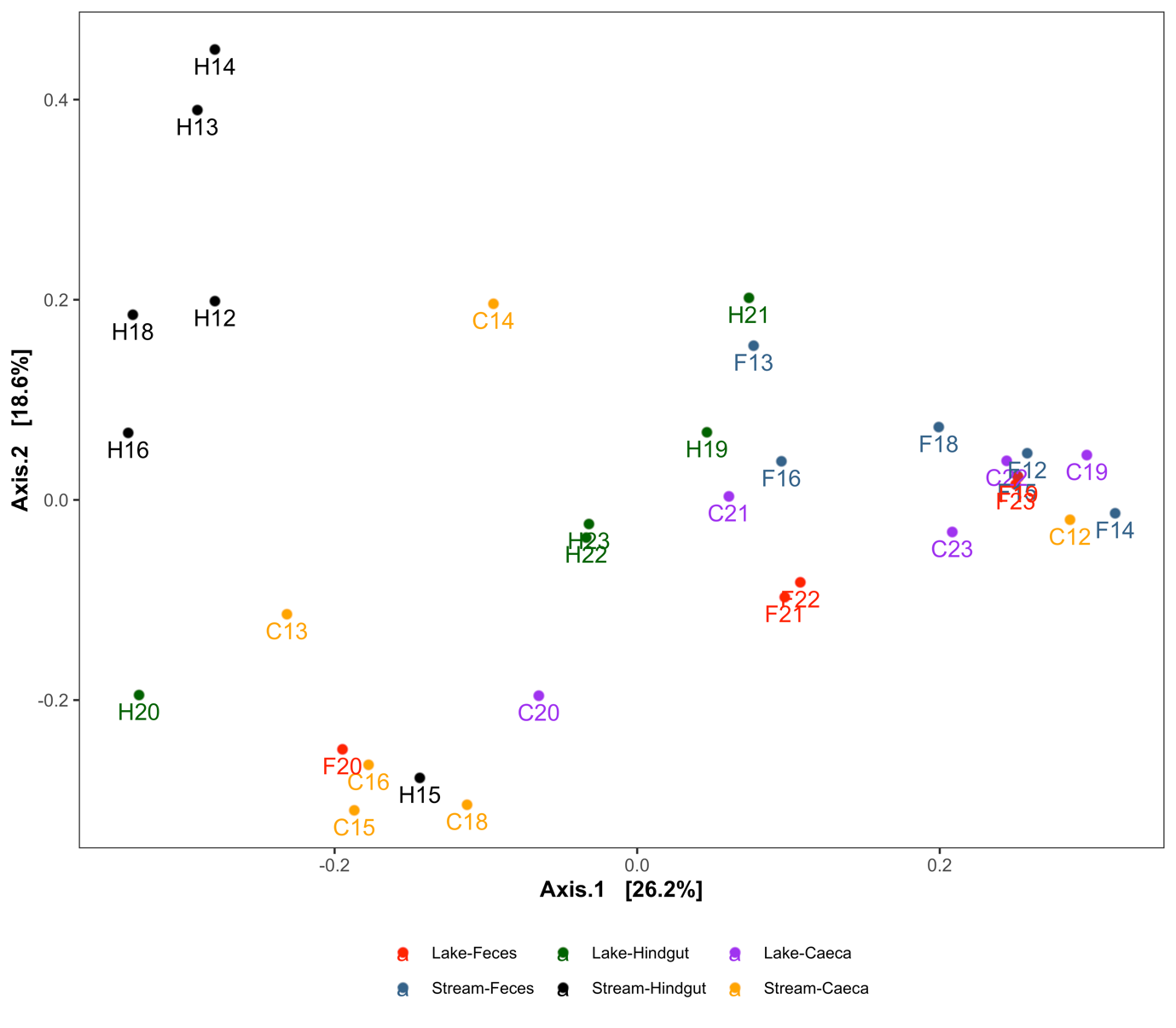


Figure S2.2 PCoA based on **weighted UniFrac** distance matrix. Only common ZOTUs present in feces, caeca and hindguts are taken into account (744 ZOTUs). Each dot represents a sample, and the number label shows which individual the sample is from. Abbreviations: H = Hindgut, C = Caeca, F = Feces.


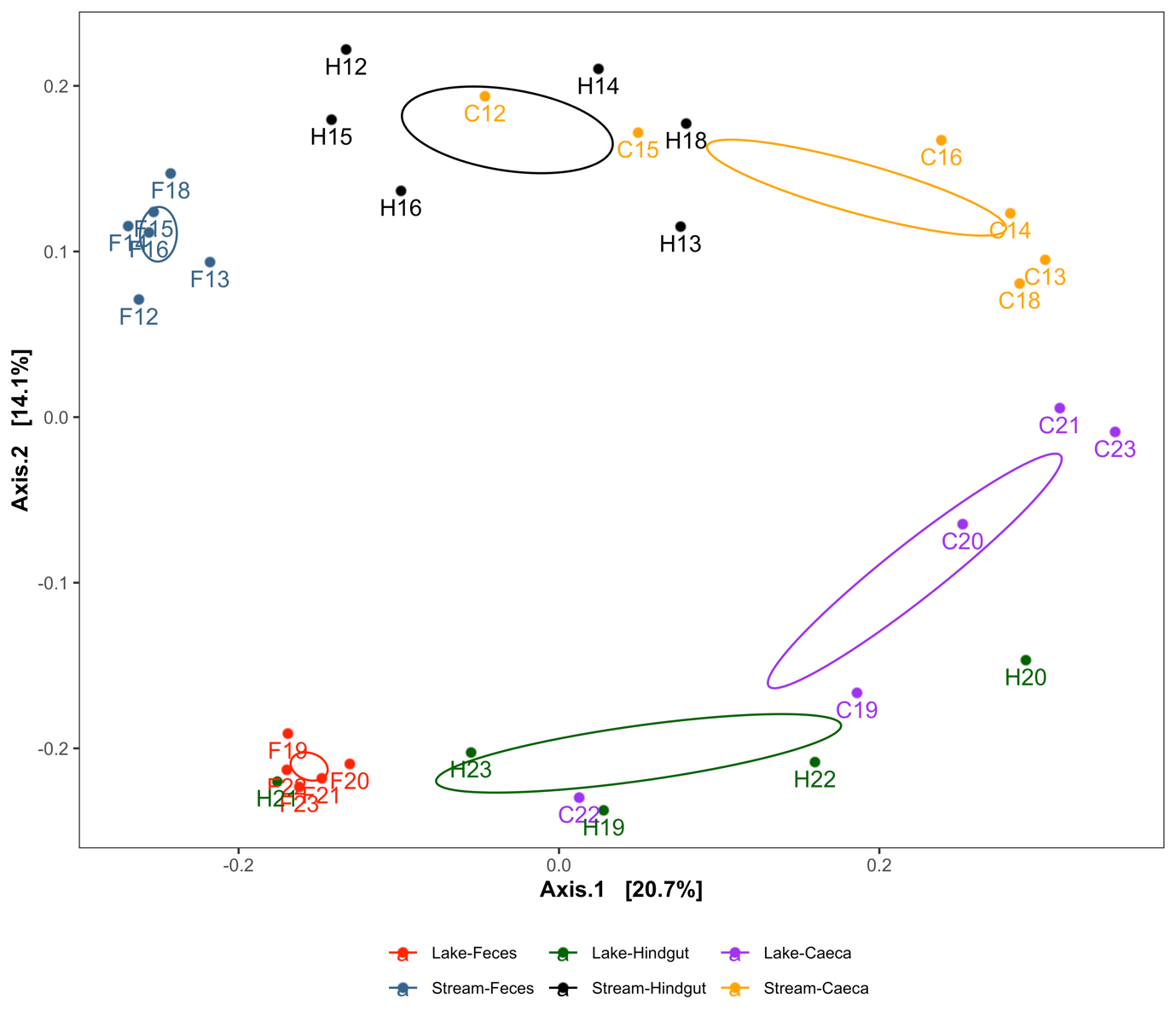


Figure S2.3 PCoA based on **unweighted UniFrac** distance matrix. Only common ZOTUs present in feces, caeca and hindguts are taken into account (744 ZOTUs). Each dot represents a sample, and the number label shows which individual the sample is from. Abbreviations: H = Hindgut, C = Caeca, F = Feces.


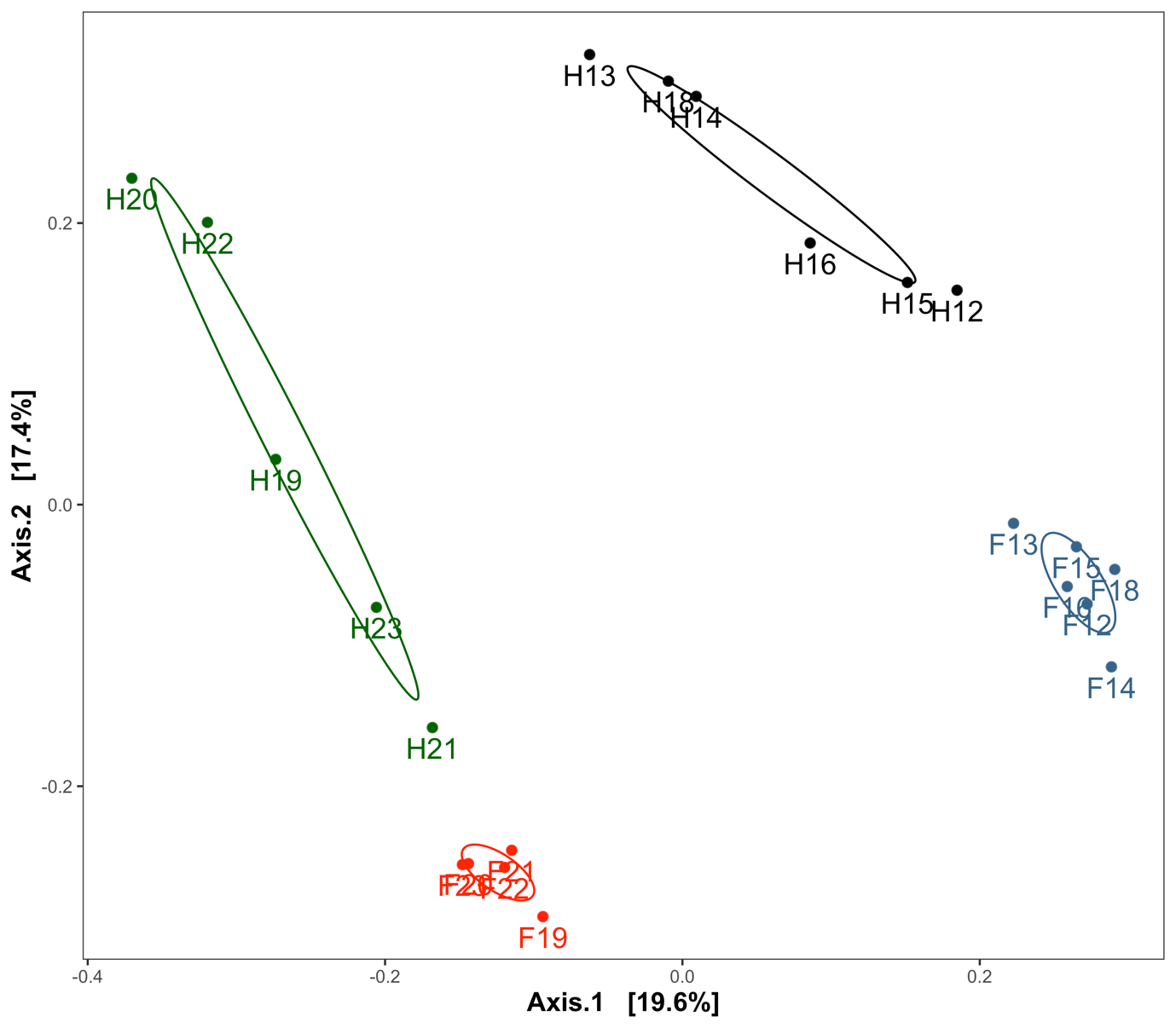


Figure S2.4 PCoA based on **unweighted UniFrac** distance matrix. **Only feces and hindgut samples are shown here.** Each dot represents a sample, and the number label shows which individual the sample is from. Abbreviations: H = Hindgut, F = Feces.

SI 3 Supplementary Tables

Table S3.1 Pairwise Comparisons of Shannon Diversity of different sample types (significance level acquired from pairwise Wilcoxon rank sum test; p values adjusted using Benjamin & Hochberg method)

| Sample Type | Lake-Caeca | Lake-Feces | Lake-Hindgut | Stream-Caeca | Stream-Feces |
| --- | --- | --- | --- | --- | --- |
| Lake-Feces | 0.587 | - | - | - | - |
| Lake-Hindgut | 0.587 | 0.226 | - | - | - |
| Stream-Caeca | 0.209 | **0.043*** | 0.411 | - | - |
| Stream-Feces | 0.209 | 0.242 | **0.032*** | **0.032*** | - |
| Stream-Hindgut | **0.043*** | **0.032*** | 0.176 | 0.699 | **0.032*** |

Table S3.2 Results of Permutational multivariate analysis of variance (adonis function). Test is based on **unweighted** UniFrac distances and 99999 permutations. Only common ZOTUs present in feces, caeca and hindguts are taken into account (744 ZOTUs).

|  | Df | Sums Sqs | Mean Sqs | F.Model | R2 | Pr(>F) |
| --- | --- | --- | --- | --- | --- | --- |
| Habitat | 1 | 0.4405 | 0.44051 | 3.4590 | 0.07727 | 0.00130*** |
| Tissue | 2 | 1.2607 | 0.63036 | 4.9496 | 0.22115 | 0.00001*** |
| Habitat:Tissue | 2 | 0.5610 | 0.28049 | 2.2025 | 0.09841 | 0.00509** |
| Residuals | 27 | 3.4386 | 0.12735 |  | 0.60317 |  |
| Total | 32 | 5.7008 |  |  | 1.00000 |  |

Table S3.3 Results of Permutational multivariate analysis of variance (adonis function). Test is based on **unweighted** UniFrac distances and 99999 permutations. Only common ZOTUs present in feces, caeca and hindguts are taken into account (744 ZOTUs).

|  | Df | Sums Sqs | Mean Sqs | F.Model | R2 | Pr(>F) |
| --- | --- | --- | --- | --- | --- | --- |
| Habitat | 1 | 0.8455 | 0.84545 | 456272 | 0.13065 | 0.00001*** |
| Tissue | 2 | 1.2357 | 0.61786 | 4.1124 | 0.19096 | 0.00001*** |
| Habitat:Tissue | 2 | 0.3335 | 0.16675 | 1.1098 | 0.05153 | 0.2617 |
| Residuals | 27 | 4.0566 | 0.15024 |  | 0.62686 |  |
| Total | 32 | 6.4713 |  |  | 1.00000 |  |

Table S3.4 Differentially enriched bacterial taxa (genus level) between stream caeca microbiome and the lake caeca microbiome. Log2FoldChange > 0 indicates higher abundance in stream caeca microbiome. Unidentified taxa are marked with asterisks.

| Bacterial Genus | baseMean | log2FoldChange | lfcSE | padj |
| --- | --- | --- | --- | --- |
| *Chthoniobacter* | 219.62 | -26.78 | 3.01 | 1.18E-16 |
| *Latescibacteria** | 145.07 | -26.28 | 3.01 | 3.49E-16 |
| *Pirellula* | 132.97 | -26.16 | 3.01 | 3.73E-16 |
| *Chloroplast** | 91.73 | -25.65 | 3.01 | 1.29E-15 |
| *Blastopirellula* | 75.46 | -25.38 | 3.02 | 2.31E-15 |
| *Ethanoligenens* | 52.69 | -24.89 | 3.02 | 7.83E-15 |
| *Cyanobacteria** | 49.18 | -24.80 | 3.02 | 8.97E-15 |
| *Cyanobacteria** | 38.25 | -24.37 | 3.02 | 2.61E-14 |
| *Cytophagales** | 36.90 | -24.23 | 3.02 | 3.36E-14 |
| *Candidatus Azambacteria** | 36.69 | -24.18 | 3.02 | 3.51E-14 |
| *Armatimonas* | 46.97 | -24.10 | 3.02 | 4.01E-14 |
| *Methylomonas* | 42.67 | -23.78 | 3.02 | 8.00E-14 |
| *Runella* | 133.74 | -10.57 | 3.01 | 3.54E-03 |
| *Rickettsiales Incertae Sedis** | 65.74 | -10.40 | 3.01 | 4.46E-03 |
| *Chloroplast** | 1339.51 | -7.09 | 2.08 | 4.93E-03 |
| ***Wolbachia*** | 4318.07 | -7.07 | 2.39 | 2.12E-02 |
| *Saprospiraceae** | 58.79 | -6.54 | 2.25 | 2.47E-02 |
| *Bdellovibrio* | 360.97 | -5.81 | 1.69 | 4.59E-03 |
| *Hyphomicrobiaceae** | 39.38 | 8.74 | 2.75 | 1.08E-02 |
| *Alteromonadales** | 146.41 | 9.01 | 2.98 | 1.81E-02 |
| *Finegoldia* | 34.81 | 9.12 | 3.04 | 1.89E-02 |
| ***Candidatus Hepatoplasma*** | 205805.19 | 10.40 | 2.90 | 2.74E-03 |
| *Flexibacter* | 33.66 | 23.24 | 3.04 | 4.03E-13 |
| *Sphaerotilus* | 318.38 | 26.37 | 2.72 | 1.26E-19 |

Table S3.5 Number of reads of *Candidatus Hepatincola, Candidatus Hepatoplasma*, and *Wolbachia* in each sample (after rarefaction). Number in bold indicates >= 50 reads. Abbreviation in SampleID: F = Feces, H = Hindgut, C = Caeca.

| SampleID | *Candidatus Hepatincola* | *Candidatus Hepatoplasma* | *Wolbachia* |
| --- | --- | --- | --- |
| C12 | 0 | 4 | 2 |
| C13 | 12 | 0 | 9 |
| C14 | 0 | **50362** | 3 |
| C15 | 2 | 3 | 18 |
| C16 | 0 | 11 | 17 |
| C18 | 0 | 0 | 0 |
| C19 | 2 | 6 | 0 |
| C20 | 0 | 0 | 7 |
| C21 | 0 | 0 | 35 |
| C22 | 0 | **55** | **21757** |
| C23 | **50** | **2273** | **77** |
| F12 | 43 | 12 | **71** |
| F13 | 0 | 0 | 13 |
| F14 | 3 | 1 | 25 |
| F15 | 0 | 0 | 4 |
| F16 | 0 | 3 | 0 |
| F18 | 0 | 0 | 9 |
| F19 | **95** | 0 | 3 |
| F20 | 45 | 0 | 8 |
| F21 | **60** | 0 | 22 |
| F22 | 10 | 0 | 3 |
| F23 | 19 | 47 | 11 |
| H12 | 0 | 1 | 7 |
| H13 | 0 | 0 | 4 |
| H14 | 0 | 9 | 8 |
| H15 | 9 | 0 | 2 |
| H16 | 0 | 0 | 15 |
| H18 | 0 | 0 | 8 |
| H19 | 5 | 0 | 10 |
| H20 | 0 | 0 | 0 |
| H21 | 15 | 3 | 37 |
| H22 | 31 | 2 | **3708** |
| H23 | 0 | 5 | 14 |
